# Using Magnetic Nanoparticles for Gene Transfer to Neural Stem Cells: Stem Cell Propagation Method Influences Outcomes

**DOI:** 10.3390/jfb6020259

**Published:** 2015-04-24

**Authors:** Mark R. Pickard, Christopher F. Adams, Perrine Barraud, Divya M. Chari

**Affiliations:** 1 Cellular and Neural Engineering Group, Institute for Science and Technology in Medicine, Keele University, Keele, Staffordshire ST5 5BG, UK; E-Mails: m.r.pickard@keele.ac.uk (M.R.P.); c.adams@keele.ac.uk (C.F.A.); 2 Department of Physiology, Development and Neuroscience, University of Cambridge, Anatomy Building, Downing Street, Cambridge CB2 3DY, UK; E-Mail: pb379@cam.ac.uk

**Keywords:** nanoparticle, magnetofection, neural cell, stem cell, transplantation, genetic engineering

## Abstract

Genetically engineered neural stem cell (NSC) transplants offer a key strategy to augment neural repair by releasing therapeutic biomolecules into injury sites. Genetic modification of NSCs is heavily reliant on viral vectors but cytotoxic effects have prompted development of non-viral alternatives, such as magnetic nanoparticle (MNPs). NSCs are propagated in laboratories as either 3-D suspension “*neurospheres*” or 2-D adherent “*monolayers*”. MNPs deployed with oscillating magnetic fields (“magnetofection technology”) mediate effective gene transfer to neurospheres but the efficacy of this approach for monolayers is unknown. It is important to address this issue as oscillating magnetic fields dramatically enhance MNP-based transfection in transplant cells (e.g., astrocytes and oligodendrocyte precursors) propagated as monolayers. We report for the first time that oscillating magnetic fields enhanced MNP-based transfection with reporter and functional (basic fibroblast growth factor; FGF2) genes in monolayer cultures yielding high transfection *versus* neurospheres. Transfected NSCs showed high viability and could re-form neurospheres, which is important as neurospheres yield higher post-transplantation viability *versus* monolayer cells. Our results demonstrate that the combination of oscillating magnetic fields and a monolayer format yields the highest efficacy for MNP-mediated gene transfer to NSCs, offering a viable non-viral alternative for genetic modification of this important neural cell transplant population.

## 1. Introduction

Multipotent neural precursor/stem cells (NSCs) derived from the developing/adult central nervous system (CNS) or embryonic stem cells are a major transplant population, offering benefits of self-renewal and multipotentiality for cell replacement. Clinical transplantation trials of human fetal NSCs have been initiated including for Pelizaeus-Merzbacher disease, chronic spinal cord injury, amyotrophic lateral sclerosis, stroke, Batten’s disease (a lysosomal storage disorder) and Parkinson’s disease, with some successful outcomes [1,2,3,4,5]. Apart from cell replacement, NSCs are believed to mediate pro-regenerative mechanisms, including neurotrophic support, scavenging of toxic molecules, immunomodulatory activity and suppression of scarring reactions in injury sites (reviewed by [2]). Further, these cells show low immunogenicity, are non-tumorigenic [2] (unlike embryonic stem cells [6] and induced-pluripotent stem cells [7]) and can functionally integrate into the host neural circuitry. Notably, NSCs can migrate long distances, especially towards foci of pathology (termed 'pathotropism'), of relevance for repair of large or multifocal lesions [8]. This migratory capacity, coupled with their amenability to genetic engineering makes NSCs ideal cellular “vehicles” for delivery of therapeutic molecules (e.g., neurotrophins) to injury sites [9]. 

Genetic modification of NSCs is heavily reliant currently on viral vectors [10,11] but instances of cytotoxicity and altered cell physiology have been reported [12,13,14,15]. Additionally, requirements for costly infrastructure for large-scale virus production means there is a significant “*barrier to translation*” for therapies using virally transduced NSCs, prompting a major drive for the development of non-viral vector systems [16,17]. Magnetic nanoparticles (MNPs) [18,19,20,21,22,23] are advanced materials comprising magnetic cores overcoated with biocompatible polymers that bind/condense DNA [21,24] and have emerged as an important class of non-viral gene delivery agents in recent years. Application of static/oscillating magnetic fields can dramatically enhance MNP based gene delivery (the so called “*magnetofection*” method) [18,19,22,23] and as gene transfer relies on intrinsic cellular endocytotic mechanisms, this approach shows high safety *versus* techniques such as nucleofection and electroporation [15,25,26]. 

In experimental neurology, NSCs are propagated using two major culture formats—namely “*neurosphere*s” and “*monolayers*”—with distinct features. We recently proved that application of oscillating magnetofection technology could enhance MNP-based transfection of NSCs propagated as neurospheres [23] but the method has never been tested for NSC monolayers. This is an important issue to address as oscillating magnetofection methods can significantly improve gene delivery to adherent cultures of neural cells such as astrocytes [19] and oligodendrocyte precursor cells (OPCs) [18]. The optimal frequencies showed cell type dependence (4 Hz for OPCs and 1 Hz for astrocytes); additionally, the major differences between neural cells in terms of nanoparticle uptake and handling [27] means that data from one neural cell type cannot be extrapolated to another, so that NSC monolayers warrant independent investigation. Therefore, the aims of this study were to investigate: (i) the effects of static and oscillating magnetic fields on MNP based delivery of reporter genes (single and combinatorial gene transfer) to NSC monolayers; (ii) the survival of magnetofected NSCs and their ability to form neurospheres (*i.e.*, the optimal culture format for cell transplantation procedures) to evaluate procedural safety; and (iii) the potential of the optimized protocol for functional gene delivery.

## 2. Results and Discussion

### 2.1. MNP-Mediated Gene Delivery to NSCs: Effect of Static and Oscillating Magnetic Fields

**Figure 1:**
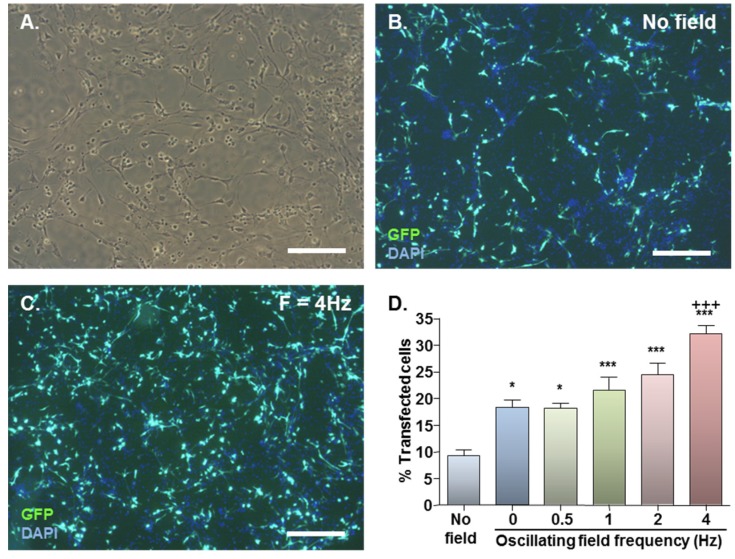
Effect of magnetofection with static (*F* = 0 Hz) and oscillating (*F* = 0.5–4 Hz) magnetic fields on transfection efficiency. (**A**) Representative phase image of monolayer cultures; (**B**) Representative double-merged image of DAPI-stained cultures at 48 h after Neuromag-mediated transfection with pmaxGFP conducted in the absence of a magnetic field; (**C**) Representative double-merged image of DAPI-stained cultures at 48 h after Neuromag-mediated transfection with pmaxGFP with an applied oscillating magnetic field of *F* = 4 Hz; (**D**) Bar chart showing proportions of transfected cells in NSC monolayers at 48 h after addition of Neuromag and pmaxGFP complexes with application of the indicated magnetic field.*P < 0.05 & ***P < 0.001 *versus* no magnetic field; ^+++^*P* < 0.001 *versus* static (*F* = 0 Hz) magnetic field; *n* = 4 cultures (one-way ANOVA and Bonferroni’s MCT). Scale bar = 200 µm in (A, B & C).

Healthy monolayer cultures of NSCs were routinely derived from dissociated neurospheres, predominantly comprising cells with elongated cell bodies with two or more processes (Figure 1A). In preliminary transfection experiments (*n* = 2 cultures) using Neuromag at 2 µL/well or 6.9 µL/mL culture medium (*i.e.*, a concentration recommended by Oz Biosciences for neuronal transfection), marked toxicity was observed in monolayers with an applied static magnetic field, evidenced by reduced cell adherence and rounding at 48 h (data not shown); this was not apparent in the absence of a magnetic field. Under an applied static magnetic field, negligible cytotoxicity was observed when the Neuromag dose was reduced to 0.62 µL/well or 2.1 µL/mL culture medium (data not shown). As procedural safety was of paramount concern in these experiments (to develop a method that is suitable for clinical translation), the latter dose was employed in all further monolayer experiments. For the same reason, a positive control, for example, a common non-viral procedure such as nucleofection was not studied here as this can result in significant loss of cell viability despite high transfection efficiency [15]. 

Basal GFP expression was detected in NSC monolayers with no applied field (Figure 1B) with a significant increase on magnetic field application (Figure 1C). In the absence of applied fields, mean transfection efficiency was 9.4% which almost doubled to 18.4% with application of a static field (Figure 1D). Oscillating fields of varying frequencies (0.5–4 Hz) also stimulated gene delivery, and the effects were frequency-dependent (Figure 1D). An oscillating field of *F* = 4 Hz yielded the highest mean transfection efficiency of 32.2% (Figure 1D), significantly higher than that obtained using static fields.

The protocol established here is a technically simple, rapid and single step procedure, compared to the multifection protocols that are required to achieve similar transfection levels in suspension cultures of NSCs [20]. Furthermore, the transfection efficiency reported here with the oscillating field of *F* = 4 Hz is three-fold that of neurospheres magnetofected under the same field conditions [23]. Magnetofection enhances transfection by increasing particle sedimentation and particle contact with cells [21]. Monolayers offer specific physical advantages over neurospheres in this context being relatively “two dimensional” and adherent rather than free-floating. The first attribute allows particle access to all cells in the system, whereas neurospheres may have inaccessible cells in their centres, reducing transfection efficiencies. This concept is supported by a decline in the extent of transfection of neurospheres with increased time in culture pre-transfection (and hence greater neurosphere diameter) [20]. The second attribute is important as the applied field source is more likely to increase particle-cell interactions in adherent cells than in suspension cell systems as field strength diminishes with increasing distance from the magnet. 

An optimal oscillation frequency of 4 Hz was established for NSC monolayers, however the mechanism/s by which oscillating magnetic fields enhance transfection, and the reasons for cell type-specific differences in optimal oscillation frequencies [18,19,23] are currently unknown. Oscillating magnetic fields may impart a lateral movement to particles, increasing their dispersion, as well as act to sediment particles. This may influence particle-plasmid complex uptake directly, by modulating the interaction of complexes with the cellular uptake machinery, and/or indirectly, via mechanical stimulation of cells, altering intrinsic endocytotic activity. In the former scenario, the rate at which coated pit formation (for receptor-mediated endocytosis) or membrane ruffling (for macropinocytosis) occurs, for example, may determine the frequency-dependence of a particular cell type, whereas in the latter scenario, factors such as cell-specific differences in the expression of mechanoreceptors, cell size or cell rigidity may be important. Nevertheless, further work is required to more fully investigate the mechanism of action of magnetic oscillation. 

In order to examine long term gene expression with this procedure, neurospheres were formed from transfected cells at 48 h *post*-transfection, and these were passaged at weekly intervals. At 7 days *in vitro* (9 days post-transfection) for the “no field” condition, around one third of neurospheres demonstrated GFP expression, but this was confined to a minor proportion of cells within spheres (Table 1). Higher proportions of GFP expressing spheres were apparent for the field-conditions, which displayed more extensive GFP expression (Table 1). Further passage revealed a marked decline in the proportions of labelled spheres after 7 days in culture (*i.e.*, at 16 days post-transfection) for all magnetic field conditions, with the extent of cell labelling within spheres classified as exclusively “low” (Table 1). Negligible GFP expression was observed after one further passage. Thus the methodology results in transient gene expression [up to 14 days; such transient expression is likely to be beneficial for conditions where repair is regulated by dynamic, temporally controlled molecular expression patterns (e.g., multiple sclerosis or spinal cord injury)]. For conditions where prolonged expression of therapeutic factors is required, it should be possible to achieve stable gene expression in NSCs by magnetofection, for example, by using plasmids that confer neomycin resistance coupled with antibiotic selection [15]. 

**Table 1:** Long-term GFP expression in neurospheres derived from transfected monolayers. Monolayers were transfected with pmaxGFP with the indicated applied magnetic fields. At 48 h, cells were detached and passaged as neurospheres; spheres were dissociated and re-plated at weekly intervals. At the indicated times, the proportion of GFP expressing neurospheres and the extent of GFP expression (based on the proportion of cells within a sphere demonstrating GFP expression) were scored; categories for the latter were “low” (≤10% cells), “moderate” (11%–50% cells) and “high” (≥51% cells).

Days *in vitro*	Field	GFP^+^ spheres (%)	Extent of GFP expression (% GFP^+^ spheres)		
			Low	Medium	High
7 (*n* = 4)	None	33.2 ± 3.0	95.5 ± 2.7	4.5 ± 2.7	0.0
	*F* = 0 Hz	57.7 ± 7.2 ^a^	89.1 ± 7.3	6.3 ± 3.6	4.9 ± 4.5
	*F* = 4 Hz	69.8 ± 1.6 ^c^	76.3 ± 11.0	16.0 ± 5.0	7.8 ± 6.9
14 (*n* = 3)	None	3.6 ± 0.6	100.0	0.0	0.0
	*F* = 0 Hz	9.6 ± 1.4 ^a^	100.0	0.0	0.0
	*F* = 4 Hz	13.4 ± 2.3 ^b^	100.0	0.0	0.0

^a^ *P* < 0.05; ^b^ *P* < 0.01; and ^c^ *P* < 0.001 *versus* no magnetic field (one-way ANOVA and Bonferroni’s MCT).

### 2.2. Safety of MNP-Mediated Gene Delivery

**Figure 2:**
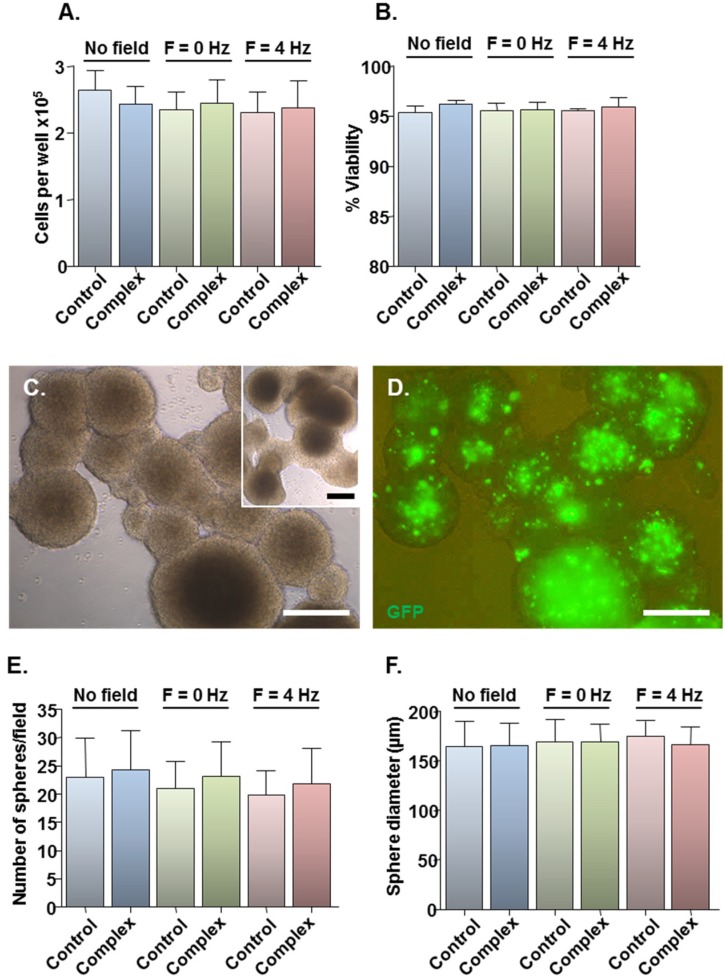
Effects of transfection protocols on cell viability and neurosphere formation. Monolayers (*n* = 4 cultures) were transfected with Neuromag-pmaxGFP complexes or with pmaxGFP only for controls, with application of the indicated magnetic fields. After 48 h, cells were detached from wells and a small proportion stained with trypan blue. (**A**) Bar chart showing the total number of cells per well. (**B**) Bar chart showing the proportion of viable cells. (**C**) Representative phase-contrast image of neurospheres formed from monolayers treated with particle/plasmid complexes; inset shows neurospheres formed from monolayers treated with plasmid only. (**D**) Fluorescence micrograph of neurospheres shown in (C), demonstrating GFP expression at 9 days post-transfection. (**E**) Bar chart showing the average sphere number per microscopic field. (**F**) Bar chart showing the average sphere diameter. Scale bar = 100 µm in (C,D).

Assessment of the safety of the developed protocols was limited to three magnetic field conditions: (i) no field, yielding the lowest transfection efficiency; (ii) static field (*F* = 0 Hz), currently the most widely used method for magnetofection; and (iii) oscillating field of *F* = 4 Hz, yielding the highest transfection efficiency. Compared with plasmid only controls, Neuromag-pmaxGFP complex addition had no effect on total cell number (Figure 2A) or viability (Figure 2B) at 48 h, irrespective of the magnetic field condition. For more stringent examination of toxicity, a neurosphere formation assay was employed in which the ability of transfected cells to form neurospheres was tested at 48 h post-transfection, with neurospheres allowed to form for 7 days. This biological assay allows for functional evaluation of cell stemness/proliferative capacity, which is key to the regenerative capacity of a transplant population such as the NSCs. Cells from treated cultures formed neurospheres (Figure 2C), which appeared morphologically similar to those from control cultures (Figure 2C, inset) with extensive GFP expression in neurospheres from transfected cultures (Figure 2D). Neurosphere number (Figure 2E) and size (Figure 2F) were similar between control and treated samples under all magnetic field conditions, indicating that the transfection protocols had no adverse effects on NSC self-renewal. In all experiments, the applied magnetic fields per se had no effect on cell number or viability (Figure 2A,B) or neurosphere formation (Figure 2E,F).

The high safety of the methods is likely to be related to the fact that cells rely on intrinsic endocytotic mechanisms for MNP uptake. From a clinical perspective, the high viability of magnetofected cells is significant, since there is a need to avoid procedures which exacerbate underlying host pathology, including the transplantation of dead or dying cells which can result in further activation of host immune responses. The ability of transfected cells to reform neurospheres is also significant in this respect, since the survival of NSCs post-transplantation may be improved by the grafting of neurospheres rather than dissociated NSCs, due in part to increased cell-to-cell survival signalling and reduced anoikis in the neurosphere transplant population [28,29,30]. Thus, oscillating field magnetofection of monolayer cultures followed by neurosphere formation (as demonstrated here for the first time), may offer the best approach for the transplantation of NSCs genetically engineered by non-viral methods, since it has the potential to ensure both high gene delivery efficacy and optimal graft survival.

### 2.3. MNP-Mediated Combinatorial Gene Delivery

**Figure 3:**
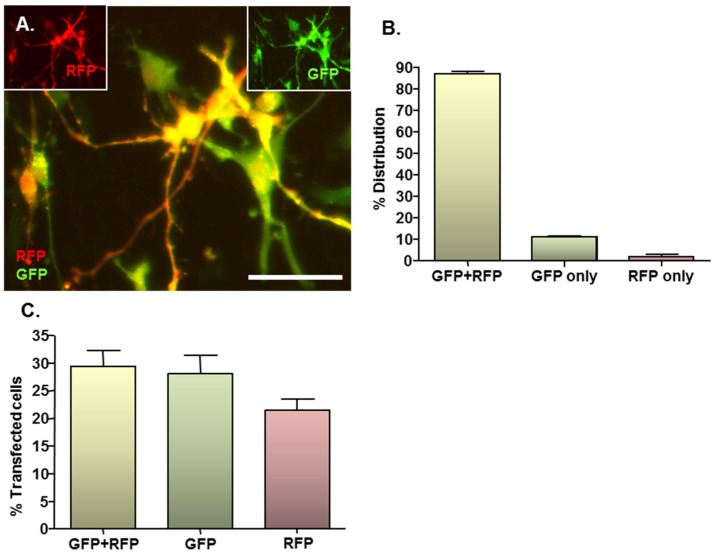
MNP–mediated combinatorial gene delivery to NSC monolayers. Cultures (*n* = 3) were magnetofected (oscillating magnetic field of *F* = 4 Hz) with complexes formed between Neuromag MNPs and either pDRE2, pmaxGFP or pDRE2 plus pmaxGFP (1:1 mix) plasmids; in all transfections, the final concentration of each plasmid was half that employed in the standard protocol. (**A**) Representative image of cells co-transfected with both plasmids. (A, insets) same field of cells in (A), showing GFP or RFP expression alone at 48 h post-transfection; (**B**) Bar chart showing the proportions of transfected cells that express GFP plus RFP, GFP alone and RFP alone after co-transfection of plasmids; (**C**) Bar chart showing transfection efficiencies for co-transfection and the corresponding single gene transfection controls. Scale bar = 20 µm in (A–C).

Given the complex nature of neural pathologies, it is unlikely that delivery of a single gene will be sufficient to augment regenerative processes in areas of neural injury, consequently combinatorial gene delivery was assessed here to more rigorously assess the clinical translational potential of the developed protocol. In co-transfected cultures, expression of both RFP and GFP could be clearly observed (Figure 3A main image and insets), with the majority of transfected cells expressing both reporter proteins. In all cases, co-transfected cells expressed normal cellular and nuclear morphologies with no evidence of cell rounding or loss, suggesting that combinatorial delivery is safe. On average, 87% of transfected cells expressed RFP plus GFP, whilst 11% expressed GFP only and the remaining 2% expressed RFP only (Figure 3B). These findings were in accordance with the results of the single plasmid transfection controls, which demonstrated a tendency (*P* = 0.056; paired Students *t*-test; *n* = 3 cultures) towards a lower transfection efficacy for the RFP-encoding plasmid *versus* the GFP-encoding plasmid (21.5% ± 1.9% *versus* 29.3% ± 2.8%) (Figure 3C). This is in accordance with the observation that transfection efficiency declines with increasing plasmid size (see Section 2.4), since the RFP-encoding plasmid is larger than the GFP-encoding plasmid (4.6 kb *versus* 3.5 kb).

Therefore, the transfection procedures are applicable to combinatorial gene delivery, of relevance for a delivery of a “*biomolecule cocktail*” to enhance distinct aspects of neural regeneration such as blood vessel and axonal outgrowth, highlighting the versatility of the methods. Combinatorial gene delivery was achieved in a high proportion (87%) of transfected cells by simply mixing the two plasmids in equal proportions before adding to particles; the relative expression levels of exogenous genes in transplant populations could therefore be fine-tuned by simply varying the proportions of plasmids used. This strategy is especially pertinent when the therapeutic aim requires the combinatorial delivery of several factors with differing potencies in host tissue. 

### 2.4. MNP-Mediated Functional Gene Delivery

**Figure 4:**
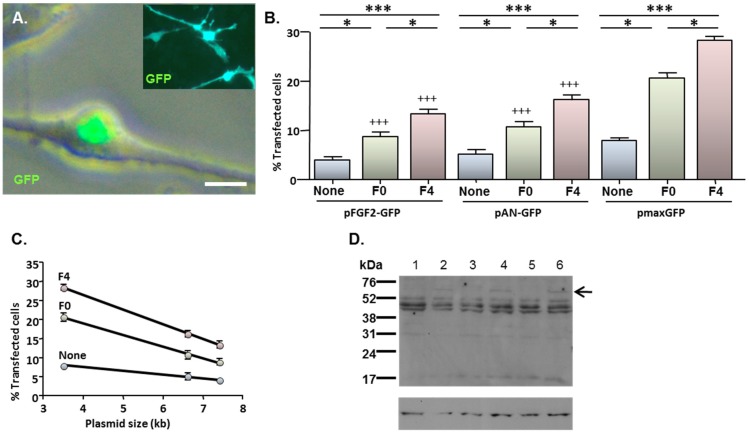
MNP-mediated delivery of a functional gene encoding FGF2—effect of magnetofection on transfection efficiency. Monolayers (*n* = 3 cultures) were transfected with Neuromag complexed with either pFGF2-GFP, pAN-GFP (control plasmid lacking the FGF2 insert) or pmaxGFP (positive control), with application of the indicated magnetic fields, then studied at 48 h post-transfection. (**A**) Representative phase and fluorescence double-merged image of cells transfected with pFGF2-GFP, demonstrating nuclear expression of GFP. Inset is a representative image of cells transfected with pAN-GFP; note that GFP expression extends throughout the cytoplasm. (**B**) Bar chart showing the proportions of transfected NSCs under no magnetic field (none), static magnetic field (F0) and oscillating magnetic field (*F* = 4 Hz; F4) conditions. *P < 0.05 and ***P < 0.001 for inter-field comparisons (indicated at top of chart) for a given plasmid; ^+++^*P* < 0.001 *versus* pmaxGFP for a given magnetic field condition (one-way ANOVA and Bonferroni’s MCT); *n* = 3 cultures. (**C**) Regression analysis demonstrating transfection efficiency is inversely related to plasmid size under no magnetic field (None; *r*^2^ = 0.994; *P* < 0.05), static magnetic field (F0; *r*^2^ = 0.998; *P* < 0.05) and oscillating (*F* = 4 Hz) magnetic field (F4; *r*^2^ = 0.999; *P* < 0.01) conditions. (**D**) Immunoblots sequentially probed with antibodies to FGF2 (top) and β-actin (loading control; bottom), demonstrating expression of a 60 kDa protein species (indicated by arrow) in extracts of cells (*n* = 3 cultures) transfected with pFGF2-GFP (lanes 2, 4 and 6) but not with pAN-GFP (lanes 1, 3 and 5); the migration of size markers is displayed on the right-hand side. Scale bar = 5 µm in (A).

Cells which had been transfected with pFGF2-GFP displayed a characteristic pattern of predominantly nuclear GFP expression (Figure 4A; main image) irrespective of the magnetic field condition. This contrasted with the more extensive cellular distribution of GFP after transfection with either pAN-GFP (Figure 4A inset) or pmaxGFP (e.g., see Figure 1B). The application of magnetic fields enhanced the transfection of all three plasmids; an oscillating field of 4 Hz yielded higher transfection efficiencies than a static field in all cases (Figure 4B). However, under each magnetic field condition, the proportions of GFP-expressing cells were lower after transfection with either pFGF2-GFP or pAN-GFP than with pmaxGFP. Notably, transfection efficiency was inversely related to plasmid size for all magnetic fields (Figure 4C). 

Immunoblotting of cell extracts with a FGF2 antibody revealed the presence of a unique band of 60 kDa in cells transfected with pFGF2-GFP when compared with cells transfected with pAN-GFP (Figure 4D); since the GFP tag contributes 26 kDa, the unique product corresponds to a FGF2 isoform of 34 kDa. The low abundance of this unique species relative to other endogenous FGF2 isoforms common to cells transfected with pAN-GFP and pFGF2-GFP plasmids (Figure 4D) is consistent with the restraints that plasmid size places on overall gene delivery efficacy (Figure 4C), with only a minor proportion of cells (between 4.1% and 13.5%; depending on magnetic field condition) achieving successful transgene (FGF2-GFP) transfection.

**Figure 5:**
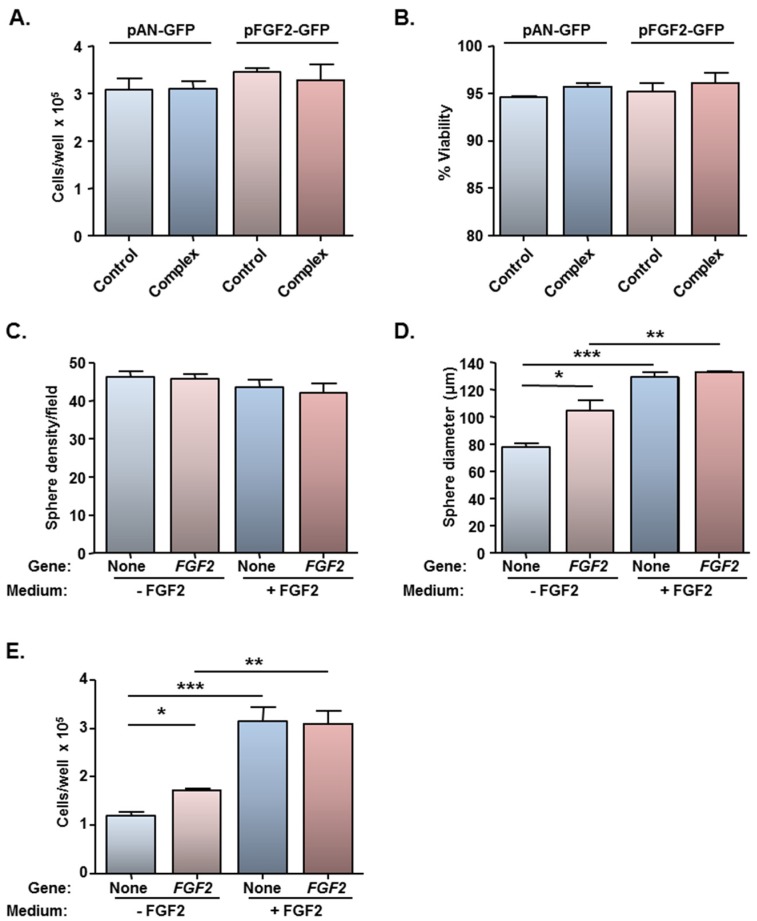
Magnetofection of a plasmid encoding FGF2 has no effect on cell viability and stimulates cell proliferation in the absence of exogenous FGF2. Monolayers (*n* = 3 cultures) were transfected with Neuromag complexed with either pFGF2-GFP or pAN-GFP (control plasmid), with application of an oscillating (*F* = 4 Hz) magnetic field. At 48 h post-transfection, cells were detached, counted (to assess cytotoxicity) and allowed to form neurospheres in culture medium with/without exogenous FGF2. (**A**) Bar chart of total cell count and (**B**) cell viability prior to neurosphere formation; (**C**) Bar chart showing neurosphere number and (**D**) size at 96 h after plating in neurosphere medium ± exogenous FGF2. *P < 0.05, **P < 0.01 and ***P < 0.001 (one-way ANOVA and Bonferroni’s MCT). (**E**) Bar chart showing total cell count after dissociation of neurospheres at 144 h after plating in neurosphere medium ± exogenous FGF2. *P < 0.05, **P < 0.01 and ***P < 0.001 (one-way ANOVA and Bonferroni’s MCT).

In agreement with earlier findings, for all three plasmids, microscopy revealed no obvious adverse effects on cell morphology and cell adherence for cells treated with complexes compared with cells treated with plasmid alone. Further analysis of cells transfected under oscillating (*F* = 4 Hz) magnetic field conditions, *i.e.*, conditions which yield the highest transfection efficiencies, revealed normal total counts and cell viability at 48 h after delivery of either pFGF2-GFP or pAN-GFP (Figure 5A,B), consistent with findings for pmaxGFP.

In order to address if the low level of FGF2 gene delivery was of functional significance, a bioassay was performed utilising cells which had been magnetofected under the *F* = 4 Hz oscillating magnetic field condition only (yielded the highest transfection efficiency of 13.5%; Figure 4B). Neurosphere formation assays are usually conducted in the presence of exogenous EGF and FGF2, and the basis of this bioassay was to examine if the FGF2-GFP transgene could functionally substitute for exogenous FGF2, which is mitogenic for NSCs. NSCs which had been transfected with pAN-GFP or pFGF2-GFP could form neurospheres in standard neurosphere medium (NS-M) and in NS-M minus FGF2. No differences were found between the two plasmids with respect to the number of spheres formed, irrespective of the culture medium (Figure 5C), but differences were apparent for sphere size (Figure 5D). Firstly, for cells transfected with the control plasmid, larger spheres were observed in the presence of exogenous FGF2 than in its absence (Figure 5D), demonstrating that the FGF2 supply is rate-limiting for cell proliferation. While no difference was apparent between sphere size between pAN-GFP and pFGF2-GFP magnetofected cells cultured in NS-M containing exogenous FGF2 (*i.e.*, FGF2 supply is saturating for cell proliferation), the mean diameter of spheres derived from pFGF2-GFP transfected monolayers was larger (by 35%) than those formed from pAN-GFP transfected cells when cultured in the absence of FGF2 (Figure 5D). In agreement with these findings, in medium lacking FGF2, there was an increase (45%) in the total number of cells recovered from neurospheres formed from pFGF2-GFP transfected NSCs compared with those formed from NSCs transfected with pAN-GFP (Figure 5E). Thus, this bioassay confirms that, despite low transfection efficiency, oscillating field magnetofection can deliver biologically relevant amounts of a therapeutic gene (FGF2); whether such levels of NSC transfection are sufficient to promote neural regeneration *in vivo* remains to be addressed. Gene expression is transient but it should be noted that regenerative events in neurological lesions are mediated by complex and changing profiles of expression of biomolecules in injury sites, therefore transient expression of repair promoting molecules by transplant populations is a desirable outcome.

Regarding the inverse linear relationship between plasmid size and transfection efficiency, it should be noted that all transfections were conducted at a fixed Neuromag:DNA ratio of 3.5 µL/µg DNA, at which binding of DNA is maximal. The most likely explanation for this relationship therefore, is that as plasmid size increases, less copies of the encoded gene will be complexed with the particles. Under these conditions, use of plasmids of the minimum size necessary to meet the desired objective is required, in order to achieve maximal gene delivery. For example, the pFGF2-GFP vector encodes neomycin resistance and carries a GFP tag, neither of which are required for transient gene expression *in vivo*. Substitution of the *gfp* sequence (size *ca.* 710 bp) in pmaxGFP with the human FGF2 cDNA sequence (size 867 bp) would produce a plasmid of ca. 3.7 kb, potentially resulting in a doubling of transfection efficiency to *ca.* 25%. A similar approach should suffice to maximize delivery of genes encoding most neurotherapeutic growth factors whose open reading frames are typically <1.2 kb in size. Our regression analyses predict an upper size limit to plasmid delivery with Neuromag particles, highlighting the need for the design of transfection-grade MNPs with increased payload capacity, to deliver plasmids with large cDNA inserts or of high complexity (e.g., contain elements for regulated expression of inserts). This could be achieved, for example, by use of alternative polymers with higher DNA binding capacity, larger particles or altered particle geometries offering greater surface area for DNA binding. 

Currently, the most popular methods to achieve gene transfer to NSCs rely on viral transduction, particularly based on the use of lentiviruses and retroviruses (for example, [31,32,33,34]). Viral approaches can be time-consuming and technically complex. Whilst recent advances in viral technology have seen increased safety profiles and scalable manufacture protocols introduced, viral systems still have their associated disadvantages in terms of safety and production scale-up [35] which impacts their suitability for clinical cell therapies. By contrast, oscillating field magnetofection as demonstrated here, represents a technically simple, quick and versatile method, which could potentially be incorporated into pre-existing automated systems, to transfer genetic material into target transplant cell populations. At non-toxic doses, MNPs outperform most other non-viral methods, notably electroporation and lipofection [12,25,26,36,37]. Whilst highly competitive with nucleofection, it should be noted that substantial loss of NSC viability can occur with the latter [25], which is undesirable for achieving optimal graft survival. The technical methods used require minimal cell manipulation and limited specialist infrastructure, implying that these can be easily adopted by other workers in the field, for a wide spectrum of therapeutic biomolecules, neural cell types and pathologies. Plasmid DNA is easy to produce in large quantities, handle and store [38] and MNPs are already in use in the clinic as contrast agents [39], therefore the potential for scale-up for clinical applications appears realistic. 

## 3. Experimental Section 

### 3.1. Reagents

Cell culture reagents/plastics were from Fisher Scientific (Loughborough, UK). Human recombinant basic fibroblast growth factor (FGF2) and epidermal growth factor (EGF) were from Sigma (Poole, Dorset, UK) and R&D Systems Europe Ltd (Abingdon, UK), respectively. The magnefect-nano 24-magnet array system was purchased from nanoTherics Ltd (Stoke-on-Trent, UK) and comprises horizontal arrays of NdFeB magnets (grade N42) on which 24-well culture plates are placed. Neuromag MNPs were from Oz Biosciences (Marseilles, France), pmaxGFP plasmid (size 3.5 kb; encodes green fluorescent protein [GFP]) was from Amaxa Biosciences (Cologne, Germany) and pCMV-DsRed-Express2 plasmid (herein termed pDRE2; size 4.6 kb; encodes red fluorescent protein [RFP]) was from Clontech (Saint-Germain-en-Laye, France). Plasmid (pCMV6-FGF2-GFP; herein termed pFGF2-GFP) encoding the open reading frame of human FGF2 with a carboxy-terminal turboGFP tag (insert size 867 bp; total size 7.4 kb), control plasmid pCMV6-AN-GFP (herein termed pAN-GFP; size 6.6 kb) and anti-FGF2 antibody (clone 3D9) were all from OriGene Technologies (Rockville, MD, USA). Vectashield mounting medium plus/minus 4´, 6-diamidino-2-phenylindole (DAPI) was from Vector Laboratories (Peterborough, UK). The care and use of all animals used in the production of cell cultures was in accordance with the Animals Scientific Procedures Act of 1986 (UK).

### 3.2. NSC Culture

NSC cultures were established from the subventricular zone of neonatal CD1 mice and routinely propagated as neurospheres [20]. To prepare NSC monolayers, neurospheres (passages 2–3) were dissociated with accutase-DNase I, cells resuspended at 3 × 10^5^ cells/ml monolayer culture medium (herein termed ML-M; comprises a 1:1 mix of DMEM:F12 containing 1% N2 supplement, 50 U/mL penicillin, 50 μg/mL streptomycin, 4 ng/mL heparin, 20 ng/mL FGF2 and 20 ng/mL EGF), then replated on polyornithine/laminin-coated, acid-washed coverslips in 24-well plates (0.4 mL suspension/well) and cultured at 37 °C in 95% air:5% CO_2_. 

### 3.3. MNP-Mediated Transfection of Monolayers

#### 3.3.1. Single Gene Delivery (Reporter and Functional Genes)

At 24-48 h post-plating, medium was replaced with fresh ML-M (0.225 mL) before addition of transfection complexes. To prepare the latter, 176 ng plasmid was diluted with 75 µL DMEM:F12 (1:1) base medium, added to 0.62 µL Neuromag and carefully mixed, corresponding to a Neuromag:DNA ratio of 3.5 µL/µg (at which Neuromag: DNA binding is maximal [19]). After 20 min, the mix was added drop-wise to cells whilst gently swirling the plate. Controls were treated with an identical concentration of plasmid only. Plates were returned to the incubator, and exposed to the desired magnetic field using the magnefect-nano oscillating magnetic array system, with a 24-magnet array (NdFeB, grade N42; field strength of 421 ± 20 mT). The array moves laterally with oscillation frequency and amplitude controlled via a computerised motor. Oscillating fields of frequencies *F* = 0.5, 1, 2 and 4 Hz were applied (amplitude = 0.2 mm), and a static magnetic field applied by setting *F* to 0 Hz. Field application was for 30 min, with incubation in the absence of a field for a further 30 min. Medium was replaced with fresh ML-M (0.4 mL) and cells cultured for a further 47 h. To assess functional gene delivery, the plasmid pFGF2-GFP was used in an identical protocol, but experiments were limited to three magnetic field conditions: none, static and oscillating (*F* = 4 Hz). Control plasmids studied in these experiments were: pAN-GFP (lacks the FGF2 cDNA insert) and pmaxGFP (positive control). 

#### 3.3.2. Combinatorial Gene Delivery

The ability of Neuromag to mediate combinatorial gene delivery was assessed using the plasmids pDRE2 and pmaxGFP, in a co-transfection protocol, with an applied oscillating magnetic field of *F* = 4 Hz. The protocol was similar to that for single gene delivery, except 88 ng of each plasmid was diluted with 75 µL base medium before adding to 0.62 µL Neuromag. Thus, the Neuromag:total DNA ratio was maintained at 3.5 µL/µg DNA, but the final plasmid concentration were halved. Therefore, single gene transfection controls using only 88 ng of each plasmid for complex formation were included.

### 3.4. Assessment of Transfection Efficiency, Toxicity and Proliferative Capacity

At 48 h post-complex addition, monolayers were washed (3X) with phosphate-buffered saline (PBS), fixed [4% paraformaldehyde in PBS, 20 min, room temperature (RT)] and washed again (PBS, 3X). Transfection efficiency was determined by fluorescence microscopy (Section 3.6). At 48 h post complex addition, monolayers were washed with PBS and cells detached by accutase-DNase I treatment. To assess cytotoxicity, cells were resuspended in ML-M (50 µL/well), mixed with trypan blue (0.2%), and viable cells counted using a Neubauer chamber and light microscopy; total cells per well and percentage cell viability were calculated. Proliferative capacity was assessed using a neurosphere formation assay: detached cells were resuspended at 1 × 10^5^ cells/ml in NS-M (3:1 mix of DMEM:F12 containing 2% B-27 supplement, 50 U/mL penicillin, 50 μg/mL streptomycin, 4 ng/mL heparin, 20 ng/mL FGF2 and 20 ng/mL EGF) and plated in 24-well suspension cell plates (0.5 mL suspension/well). Cells were cultured for 7 days, with medium additions every 2–3 days, and intact neurospheres photographed to estimate sphere size, number and proportions of transfected spheres (Section 3.6). To assess long-term gene expression, neurospheres were dissociated (accutase-DNase I), replated at weekly intervals and scored for the proportions of labelled spheres and for the extent of transfection. A neurosphere assay was also used to assess functional effects of FGF2 gene delivery on NSCs, since FGF2 is mitogenic for NSCs. The standard neurosphere medium contains exogenous FGF2, which may mask mitogenic effects of transgenic FGF2, therefore neurospheres were formed in standard NS-M and in NS-M minus FGF2. Sphere number and size were determined at 96 h, and neurospheres were dissociated at 144 h for cell counting. 

### 3.5. Immunoblotting

Transfected NSCs were detached (accutase:DNase I), washed twice with ice-cold PBS, then protein extracted with RIPA buffer containing 1% (v/v) protease inhibitor cocktail (80 µL buffer/10^6^ cells) for 30 min on ice with periodic vortexing. Samples (15 µL) were denatured with an equal volume of 2X Laemmli buffer by boiling (10 min), centrifuged (10,000 *g*; 10 min; 4 °C) and supernatants electrophoresed (12% Tris-HCl Ready gels); protein was electrotransferred in Towbin buffer to Immobilon-P membrane. Immunodetection was by the standard protocol supplied with the ECL Western blotting detection reagent, using 5% non-fat dry milk as blocker. Blots were probed with FGF2 primary antibody at 1:1000 dilution, and secondary antibody at 1:1000 dilution, stripped then reprobed with β-actin (loading control) primary antibody at 1:10000 dilution and secondary antibody at 1:1000 dilution. All blots were exposed to Hyperfilm-ECL (preflashed to 0.05 optical density units above background), and images captured using a Bio-Rad GS-800 scanner.

### 3.6. Microscopy and Image Analysis

Fluorescence microscopy of monolayers and tissue slices was performed using an AxioScope A1 microscope equipped with an Axio Cam ICc1 digital camera and AxioVision software (release 4.7.1, Carl Zeiss MicroImaging GmbH, Goettingen, Germany). Phase-contrast and fluorescence microscopy of live neurospheres was performed using a Leica DM IL LED inverted microscope equipped with a FC420C digital camera and Leica Applications Suite software version 3.4.0 (Leica Microsystems, Wetzlar, Germany). Images were merged using Adobe Photoshop CS3 (version 10.0.1) prior to quantification.

The efficiency of single gene transfection was determined from double merges of DAPI and GFP/RFP as appropriate; a minimum of 200 cells at ×200 magnification were scored. The efficiency of combinatorial gene delivery was assessed from triple-merges of DAPI, GFP and RFP images; ≥50 transfected cells at ×200 magnification were scored. Sphere size/number was determined from phase-contrast micrographs and the average sphere size/number per culture determined. Proportions of transfected neurospheres were determined from double-merges of phase-contrast and GFP images; a minimum of three microscopic fields at ×100 magnification (≥50 neurospheres in total) were assessed. Neurospheres were further scored for the extent of transfection, based on the proportion of cells within a sphere demonstrating GFP expression; categories were “low” (≤10%), “moderate” (11%–50%), and “high” (≥51%). 

### 3.7. Statistical Analysis

Data are expressed as mean ± SEM. A paired Student’s *t*-test was used to compare transfection efficiencies for pmaxGFP and pDRE2 controls in the combinatorial gene delivery experiment; all other data were analyzed by a one-way ANOVA and Bonferroni’s multiple comparison test (MCT). Statistical analyses were performed using GraphPad Prism 4 for Windows software (version 4.03). The numbers of experiments (*n*) relate to the number of NSC cultures, each generated from a different litter.

## 4. Conclusions 

Oscillating magnetic fields utilized with MNPs offer significant advantages for safe and efficient transfection of NSCs propagated as monolayers (in contrast to NSC propagation in the suspension neurosphere format where transfection levels are considerably lower). The methodology can be used both for delivery of multiple and neurotherapeutic genes, highlighting the relevance of the approach to genetically augmenting the repair capacity of transplant populations to regenerative neurology. 
